# Growth and Biochemical Responses of Potato Cultivars under In Vitro Lithium Chloride and Mannitol Simulated Salinity and Drought Stress

**DOI:** 10.3390/plants10050924

**Published:** 2021-05-06

**Authors:** Farooq Abdul Sattar, Bahget Talat Hamooh, Gordon Wellman, Md. Arfan Ali, Saad Hussain Shah, Yasir Anwar, Magdi Ali Ahmed Mousa

**Affiliations:** 1 Department of Arid Land Agriculture, Faculty of Meteorology, Environment and Arid Land Agriculture, King Abdulaziz University, Jeddah 21589, Saudi Arabia; bhamooh@kau.edu.sa (B.T.H.); arfanhort1978@gmail.com (M.A.A.); 2 Agricultural Research Institute, University of Swat, Mingora 19130, Pakistan; 3 Division of Biological and Environmental Sciences and Engineering, King Abdullah University of Science and Technology, Thuwal 23955-6900, Saudi Arabia; gordon.wellman@kaust.edu.sa; 4 Sher-e-Bangla Agricultural University, Dhaka 1207, Bangladesh; 5 Institute of Biotechnology and Genetic Engineering, The University of Agriculture, Peshawar 25120, Pakistan; bukharibkt@gmail.com; 6 Department of Biological Sciences, Faculty of Science, King Abdulaziz University, Jeddah 21589, Saudi Arabia; yasirpcsir2006@gmail.com; 7 Department of Vegetables, Faculty of Agriculture, Assiut University, Assiut 71526, Egypt

**Keywords:** *Solanum tuberosum* L., micropropagation, growth, abiotic stress, tolerance, antioxidants

## Abstract

Globally, drought and salinity stress critically constrain potato (*Solanum tuberosum* L.) production. Considering the impact of these stresses on crops and increasing food demand, insight into both tolerance and susceptibility is essential. The present study screens two potato cultivars, BARI-401 and Spunta, for their tolerance to simulated salinity and drought by in vitro LiCl and mannitol exposure. Plantlets treated with a range of LiCl (0, 10, 30, and 40 mM) and mannitol (0, 50, 100, 200, and 250 mM) concentrations were biochemically and physiologically characterized to assess their tolerance capacity. Shoot number, shoot length, root number, and root length were affected in both cultivars under higher LiCl and mannitol concentrations, even though Spunta was able to better maintain a higher shoot length under the 40 mM of LiCl and 250 mM of mannitol compared to BARI-401. The total phenol contents (TPC) in both cultivars were increased at the highest treatment concentration and the total flavonoids content (TFC) was decreased in BARI-401 as compared to Spunta. Higher free radical scavenging capacity (FRSC, low IC_50_ value) was recorded in Spunta as compared to BARI-401 with increasing treatment concentrations, which supports the high antioxidant capacity of Spunta. An inverse correlation between polyphenol oxidase (PPO) and TPC was noted in both cultivars. Peroxidase dismutase (POD) activity was increased significantly in both cultivars for all treatments, but activity was highest overall in Spunta. These physiological and biochemical analyses of both cultivars suggest that cultivar Spunta is more tolerant to salinity and drought stress. Further open-field experiments are required to confirm these results.

## 1. Introduction

In terms of global production, potato (*Solanum tuberosum* L.) is ranked fourth after wheat, corn and rice [1,2], grown in over 158 countries, and feeds over a billion people worldwide [3]. The estimated total world production of potatoes is 388 million tons, on an area of approximately 19.3 million hectares, with a total worth of USD 92 billion [2]. Potato is considered highly to moderately sensitive to salinity [4] and drought [5]. Due to this, the yield of potato is limited in arid and semi-arid regions, despite being an important crop in these regions [6,7]. 

Major constraints to crop productivity are abiotic stresses such as salinity, drought, heat, frost, and mineral toxicities [8] with drought and salinity the most prominent and are often in combination [7]. Salinity affects almost 20% of irrigated land [9] and is predicted to decrease arable land by up to 50% by 2050 [10]. Drought is estimated to decrease potato yield globally by 32% between 2040 and 2069 [11]. Considering the impact of salinity and drought on crops, there is a growing need to identify tolerant cultivars to fulfill increasing demands for food globally.

Drought and salinity stress interferes with many of the plants physiological and biochemical processes: causing osmotic stress, ion imbalance and toxicity, mineral deficiency, and oxidative stress [7]. Ultimately, these conditions interact with cellular components, especially DNA, proteins, and lipids, thus negatively impacting plant growth and development [12]. High soil salinity is characterized by an excess of Na^+^ and Cl¯ ion concentration in the soil solution that then triggers osmotic and ionic stress [13,14]. The response of plant growth to salinity has two different phases. Firstly, the rapid osmotic phase, and secondly; a slower ion-specific phase. The former phase begins immediately after the exposure of the roots to salt, which impedes growth significantly, while the latter phase starts when an accumulation of salt exceeds the threshold level of the leaves, ultimately causing plant senescence [15]. 

Plants possess different biochemical and molecular mechanisms to combat abiotic stresses, such as the production of antioxidants, ion homeostasis, and an accumulation of compatible solutes [8]. Several cytotoxic reactive oxygen species (ROS), including superoxide radicals (O^2−^), hydrogen peroxide (H_2_O_2_), singlet oxygen (^1^O_2_), and hydroxyl radicals (OH^−^), are regularly produced in plant cells and are detrimental to the normal metabolism of the cell. To manage these stresses, plants have developed a complex antioxidant defense system with enzymatic like superoxide dismutase (SOD), peroxidase (POD), catalase (CAT), polyphenol oxidase (PPO), ascorbate peroxidase (APX), guaiacol peroxidase (GPX), glutathione reductase (GR), or non-enzymatic components (ascorbate, phenolic compounds, glutathione etc.) [16,17]. Variations in the level of expression of antioxidant enzymes may be found depending on the tolerance and sensitivity of genotypes [15]. Similarly, changes in the production and distribution of non-enzymatic antioxidants, such as phenolic acids and flavonoids, have been observed in response to drought and salt stress [18,19]

Selection may be applied to selecting and regenerating plants with desirable characteristics [20]. Selection agents, such as NaCl or LiCl for salt stress and polyethylene glycol (PEG), mannitol, and sorbitol for drought stress, are introduced to media to simulate salt and drought stress [7,21]. Surviving plants are selected for further study [8]. This technique has been successfully employed in many crops, including rice, potato, wheat, coconut, banana, sweet potato, alfalfa, and sugarcane [8,21,22,23]. 

The successful application of biotechnology to improve plant abiotic stress tolerance requires an extensive understanding of the biological mechanisms associated with stress tolerance [24]. However, in vitro selection, especially for salinity and drought, is important for tolerance screening and improving tolerance in crop plants. This relatively low-tech approach is feasible for developing tolerant plants in controlled environments with limited space and time [25]. This study is designed to screen two potato cultivars Spunta (yellow skin) and BAR-41 (red skin) on physiological and biochemical stress determinants. Spunta is widely cultivated in Saudi Arabia (https://www.saudi-arabia.cropscience.bayer.com/en/Crops/Potato.aspx, accessed on 6 April 2021), the climate of which is predominantly arid [26], while BARI-401 is primarily imported for consumption. The assessment of drought and salinity tolerance of these two varieties is important for advising crop selection.

## 2. Results

### 2.1. Shoot and Root Parameters

#### 2.1.1. Effect of LiCl Levels on Shoot and Root Parameters

**Figure 1:**
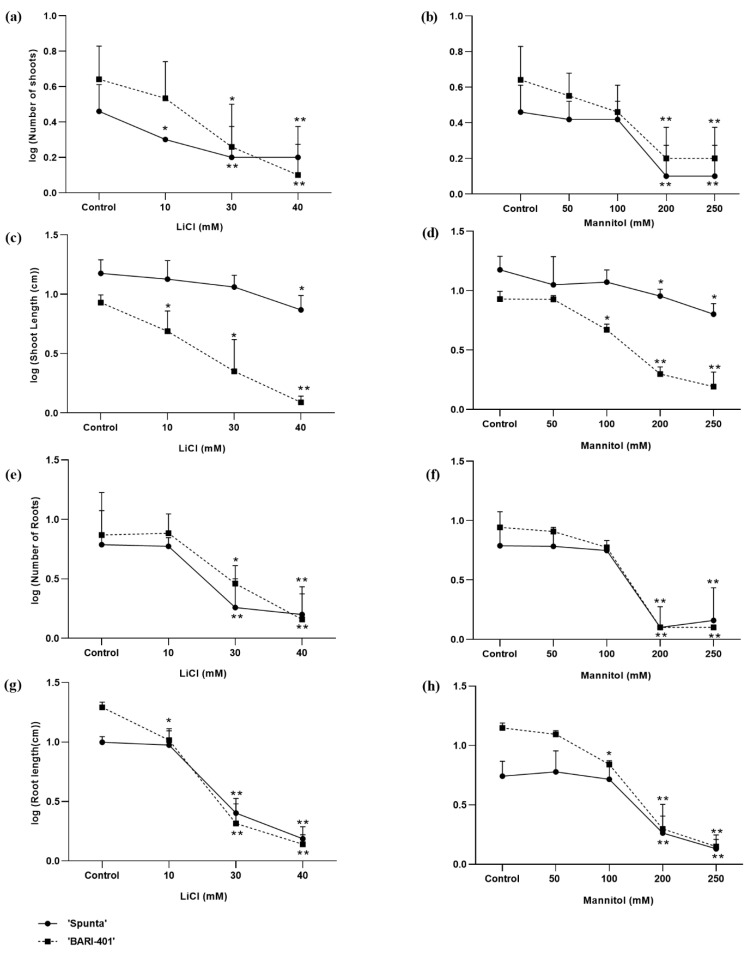
Log transformed growth parameters for BARI-401 and Spunta treated with LiCl (10, 30, and 40 mM) and mannitol (50, 100, 200, and 250 mM). (**a**,**b**): Number of shoots; (**c**,**d**): Shoot length (cm); (**e**,**f**): Number of roots; (**g**,**h**): Root length (cm). Significance between treatments and control (plantlets grown without additional LiCl or Mannitol) indicated by * (*p* < 0.05) and ** (*p* < 0.01) as determined by ANOVA with LSD.

In BARI-401, the number of shoots, shoot length and root length decreased (*p* ≤ 0.05) with LiCl concentrations of 10 mM and above, while the number of roots decreased (*p* ≤ 0.05) under 30 mM and above. Spunta maintained shoot length (*p* ≤ 0.05) until 40 mM LiCl, although it displayed decreased shoot and root number (*p* ≤ 0.01) when treated with 30 and 40 mM LiCl and reduced root length (*p* ≤ 0.05) when treated with 10 mM LiCl (*p* ≤ 0.05) (Figure 1).

#### 2.1.2. Effect of Mannitol Levels on Shoots and Roots 

The plantlets of both cultivars survived 250 mM mannitol; however, both were negatively affected. The number of shoots in both cultivars was reduced at concentrations of 200 mM and above (Figure 1b). The shoot length of BARI-401 decreased in mannitol concentrations above 100 mM while the shoot length of Spunta decreased at concentrations of 200 mM and above (*p* ≤ 0.05) (Figure 1d).

Similarly, the number of roots decreased when treated with 200 and 250 mM of mannitol in both cultivars. The root length showed a similar trend. BARI-401’s root length was reduced under 100 mM mannitol (*p* ≤ 0.05), while Spunta was able to maintain its root length until reaching 200 mM (Figure 1f,h).

### 2.2. Biochemical Analysis under Different Levels of LiCl and Mannitol

#### 2.2.1. Total Phenol Contents

**Figure 2:**
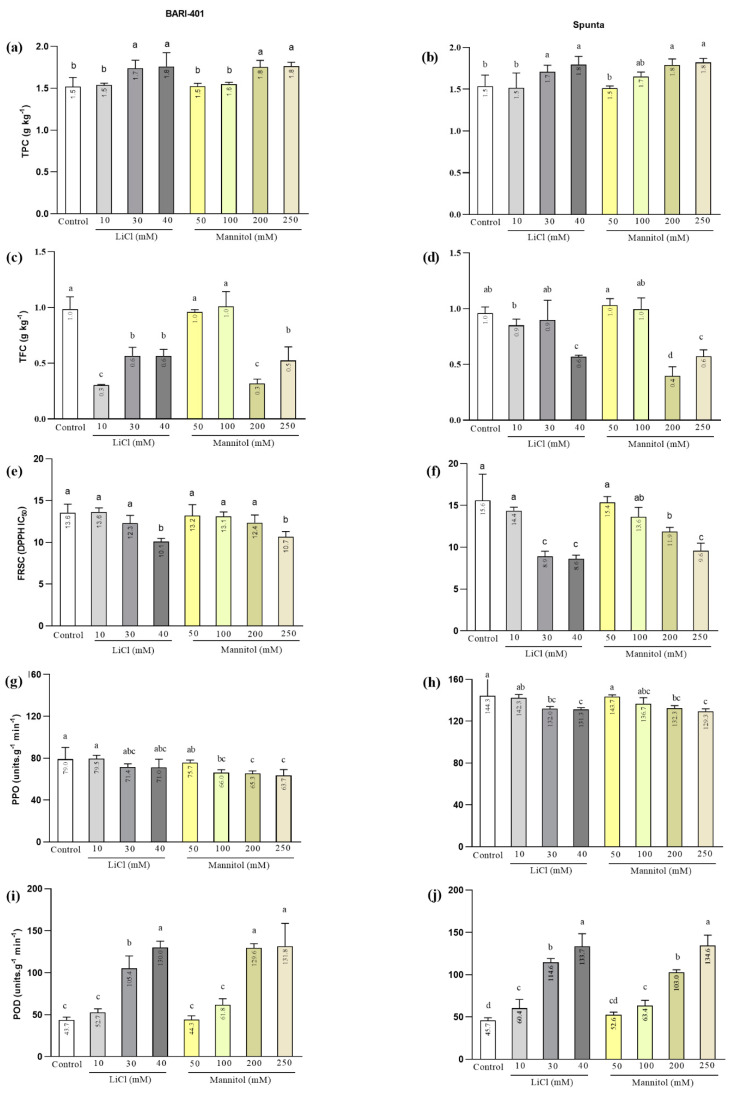
Biochemical parameters of BARI-401 and Spunta when treated with LiCl (10, 30, and 40 mM) and mannitol (50, 100, 200, and 250 mM). (**a**,**b**): total phenols concentration (TPC); (**c**,**d**): total flavonoids concentration: (**e**,**f**): free-radical scavenging capacity (FRSC); (**g**,**h**): polyphenol oxidase (PPO) specific activity; (**i**,**j**): peroxidase (POD) specific activity. Significance between treatments and control (plantlets grown without additional LiCl or Mannitol) indicated by letters, means followed by the same letter are not significantly different at (*p* < 0.05) as determined by ANOVA with LSD.

BARI-401 and Spunta displayed higher TPC (*p* ≤ 0.05) in 30 and 40 mM of LiCl and 200 and 250 mM concentrations of mannitol when compared to the controls (0 mM of LiCl or 0 mM mannitol) (Figure 2a). Similarly, Spunta displayed increased TPC when treated with 30 and 40 mM of LiCl and 200 and 250 mM of mannitol when compared to the controls, although it was trending upwards when treated with 100 mM of mannitol. (Figure 2b).

#### 2.2.2. Total Flavonoids Contents (TFC) 

The BARI-401 plantlets treated with 10, 30, and 40 mM of LiCl or 200 and 250 mM of mannitol had reduced (*p* ≤ 0.05) TFC when compared to the controls (Figure 2c). Spunta showed reduced TFC in the 40, 200, and 250 mM mannitol treatments (Figure 2d).

#### 2.2.3. Antioxidant Activity (DPPH Assay) 

The antioxidant activity of BARI-401, as measured by a DPPH free-radical scavenging assay, was increased (*p* ≤ 0.05) (lower IC_50_ value) when treated with 40 mM of LiCl and 250 mM of mannitol, although it was not affected by lower treatments (Figure 2e). Spunta antioxidant activity was higher (lower IC_50_ value) than the controls in 30 and 40 mM of LiCl and 200 and 250 mM of mannitol treatments (Figure 2f). 

#### 2.2.4. Polyphenol Oxidase (PPO)

The BARI-401 plantlets treated with 30 and 40 mM of LiCl or 100, 200 and 250 mM of mannitol showed reduced (*p* ≤ 0.05) PPO activity when compared to the control (Figure 2g). Spunta significantly reduced PPO activity at 30 and 40 mM of LiCl and 200 and 250 mM of mannitol as compared to the control (Figure 2h). Additionally, the PPO activity was higher in the Spunta compared to BARI-401 in all treatments. 

#### 2.2.5. Peroxidase (POD) 

The POD activity was increased (*p* ≤ 0.05) in the BARI-401 plantlets treated with 20 and 40 mM of LiCl and 200 and 250 mM of mannitol (Figure 2i). Spunta displayed significantly increased POD activity in treatments of 10, 30, and 40 mM of LiCl and 100, 200, and 250 mM of mannitol compared to the controls (Figure 2j).

## 3. Discussion

Screening for drought and salinity affect various biochemical and physiological processes and triggers osmotic and oxidative stress [27] that impacts plant growth and development [16]. Salts such as LiCl may be used to induce salinity stress [21] and mannitol may be used to induce drought-like conditions in plants [28]. In vitro drought and salinity stress effects have been suggested to be similar to field conditions [6]. Though it is not a direct replacement, in vitro screening of potato genotypes for salinity and drought tolerance is simpler than in vivo experiments due to the complications that arise from spatial and soil heterogeneity and climate fluctuations [29]. In the two cultivars of this study, we observed a decrease in the number of shoots, shoot length, root length, and the number of roots with an increased concentration of LiCl and mannitol in both cultivars. Reduced plant growth and abiotic stress are considered to be a negative symptom of stress [30,31] due to the reduced growth of new leaves that is associated with limited stem elongation which affects the photosynthetic capacity of the plant [32].

The number of shoots per plant also decreased with the increasing LiCl and mannitol concentrations compared to the controls in our experiment. These results are consistent with the report of Ahmed et al. (2020) [33], where they treated potato genotypes with NaCl in vitro and had no significant reduction in shoot numbers at 50 mM, but cv. innovator tolerated 100 mM of NaCl. Similarly, under limited water availability, a decreased growth pattern was observed when potato plantlets were kept under drought conditions [4]. 

Shoot length was also reduced with an increase in LiCl and mannitol concentrations in both cultivars. The reduction was more pronounced in BARI-401 when treated with 40 mM of LiCl and 250 mM of mannitol. Our results are similar to studies, that reported a reduction in shoot length of potato cultivars with an increase in NaCl concentration [33,34]. Growth parameters, such as shoot length and the number of shoots, were also observed to decrease in the potato genotypes grown on mannitol supplemented with MS media [35]. Similarly, native Chilean potato genotypes displayed reduced growth parameters when sub-cultured in modified MS media with the osmotic active compounds of mannitol and sorbitol at 100 to 300 mM concentrations [36]. 

The mean number of roots per plant was reduced as LiCl and mannitol concentration increased. The results are similar to a study of potato varieties which were screened on media supplemented with 150 mM of NaCl, with one cultivar, called Kennebec, produced the highest number of roots, leading the authors to suggest that Kennebec has an increased tolerance against salinity [33]. Similar results were recorded in the experiment of Naik and Widholm (1993) [37], where they reported the reduced development of roots when treated with 100 mM of NaCl in potato plants. 

Our results show that a reduction in root growth was more pronounced in higher treatments of LiCl and mannitol than the reduction in shoot length for both cultivars. Previous studies have indicated that potato roots and tubers are more affected than above-ground tissue when kept under drought and at 250 mM of NaCl stress in greenhouse experiments [4]. This is likely due to the fact that the roots are exposed directly to these stresses and are suggested to be the critical organs for abiotic stress tolerance in a plant [38].

Drought and salinity trigger oxidative stress and increase the flavonoid and phenol production in plants [39]. BARI-401 and Spunta show a similar response to LiCl and mannitol considering the total phenol concentration (TPC) (Figure 2a,b). Increased TPC in response to high LiCl and mannitol concentrations indicate that both cultivars possess some tolerance to these stressors. Previously, Daneshmand et al. [22] have shown that the tolerant wild potato (a relative to *Solanum acaule*) has an increased phenolic compound production in response to salinity. Similarly, increased production of phenolic compounds have been reported in wheat [40], strawberry [41], and *Cakile maritime* [42]. Similarly, assessing the TPC in two species of *Achillea* under drought found the contents to be increased under drought conditions [43]. Previously, we showed that the TPC were not altered under moderate LiCl and mannitol levels in BARI-401 and Spunta [44]. 

Stress-produced ROS are effectively scavenged by low-molecular-weight phenolic compounds and protect the cell organelles from damage [45]. We found reduced activity in the total flavonoid concentration (TFC) of both cultivars when treated with LiCl and mannitol. Previous studies support the reduction in TFC in *Simmondsia chinensis* when exposed the explants to between 50 and 500 mM of mannitol [45]. This may be due to the induction of phenolic biosynthesis under mild drought stress, whereas the enzymes related to the biosynthesis could be partially inactivated, thus resulting in low phenolic contents [46]. Moreover, the stress conditions trigger the flavonoid biosynthesis when the activity of anti-ROS enzymes start to decline [47].

BARI-401 only shows high antioxidant activity (low IC_50_) in under 40 mM of LiCl and 250 mM of mannitol, whereas Spunta increases activity in treatments of 30 and 40 mM of LiCl and 200 and 250 mM of mannitol (Figure 2e,f). Higher antioxidant activity in response to drought has been previously reported in *Salvia officinalis* [46]. Abiotic stress in plants often involves oxidative stress that triggers the production of ROS—such as superoxide radical (O^2−^), hydroxy radical (OH), hydrogen peroxide (H_2_O_2_), and alkoxy radical (RO) [48]—that then induces the antioxidant defense system [49] to safeguard plants from damage [50]. Similarly, Kim et al. [51] suggested that salinity stress may alter phenolic compound production and antioxidant activity, but critically, this depends on the plant’s sensitivity to salinity. 

Another system in plants that combats stress-induced ROS production is the enzymatic detoxification responses. ROS scavenger enzymes, such as CAT, POD, SOD, APX, and PPO activities, increase [52]; but depend on the plant’s sensitivity to abiotic stress [53]. Both cultivars in this study show a decreasing trend in PPO activity as the LiCl and mannitol concentration increase (Figure 2g,h). We observed an inverse correlation between PPO and phenolic compounds in the two potato cultivars when cultured in vitro under abiotic stress. Overall, Spunta has a higher PPO activity than BARI-401, which may help support and maintain shoot growth under stress conditions. Previous studies have reported that PPO is involved in the phenolics oxidation in plants and inverse correlation has also been observed in *Crocus sativus* [54]. Additionally, an inverse correlation trend between PPO and phenolic compounds was observed in watermelon and tomato when exposed to hot and cold stresses, with both accumulating soluble phenolic compounds due to their inhibited PPO activity under stress [55]. Similarly, an inverse correlation was also reported in *Olea europaea* (Olive) fruit, where a compound, oleuropein, plays a role in phenolics metabolism [56].

POD scavenges harmful H_2_O_2_ produced during dismutation. SOD and POD protect the stability of the cell membrane system mutually by reducing the level of membrane lipid oxidation [57]. The increasing POD activity in both cultivars was examined in this study by being placed in LiCl and mannitol treatments, with Spunta increasing at lower mannitol levels suggesting that an earlier stress response may allow it to respond more efficiently than BARI-401 to drought stress. This has been reported by us previously in a study where Spunta was found to respond differently than BARI-401 when treated with 20 mM of LiCl and 150 mM of mannitol with several biochemical and metabolomic profile changes [44]. Similarly, POD activity has been observed to increase in potato genotypes Agria, Diament, Kennebec, and Ajax when exposed to 50 mM of NaCl, while POD and CAT contribute to a ROS detoxification [58]. In another study, the potato cultivars Russett, Burbank, Desiree, and Unica displayed enhanced POD activity under heat and drought stress, while no activity was observed in the sensitive genotype Agria and there was no reaction to SOD activity in any of the genotypes [16]. This suggests that the response of antioxidant enzymes is cultivars dependent [58].

## 4. Materials and Methods

### 4.1. Plantlets and In Vitro Conditions

Potato (*Solanum tuberosum* L.) plantlets were grown from certified cultivar tuber sprouts, BARI-401 (red skin) and Spunta (yellow skin), obtained from Astra Food company, Tabuk, Saudi Arabia. After sterilization with 70% ethanol and 20% commercial bleach [59], the sprouts were cultured on autoclaved (15 min at 121 °C with 15 psi) media 50 mL of MS media [60], containing 4 gL^−1^ of phytagel, 30 gL^−1^ of sucrose, 2mgL^−1^ of 6-benzylaminopurin (BAP), 1 mgL^−1^ of indolebutyric acid (IBA), and 0.25 mgL^−1^ of gibberellic acid (GA_3_) [61] in Duran poly-carbonated tissue culture bottles (Duran® Erich-Schott-Strasse 14 95666 Mitterteich, Germany). The pH of the media was adjusted to 5.7–5.8 by using 0.1N of NaOH/HCL. To obtain a sufficient plant population for the experiments, there was a sterile monthly subculturing of the stem nodal segments. The subcultures were grown under fluorescent lighting with a 16 h photoperiod, 23 + 2 °C temperature, and 50 + 5% relative humidity.

### 4.2. Plantlets Treatment with LiCl and Mannitol

Homogenous stem nodal segments of both cultivars (BARI-401 and Spunta) were sub-cultured into standard MS media (control) or MS media containing additional LiCl (10, 30, 40, and 50 mM) [21] or mannitol (50, 100, 200, and 250 mM) [62] and grown under the lighting and temperature regime described above.

### 4.3. Shoot and Root Parameters

The shoot and root parameters of the fully developed plantlets prior to senesce were measured 50 days after subculturing. The plantlets were harvested and the roots were thoroughly washed with distilled water to remove the attached medium. The shoots were cut from the roots and the number of shoots and roots originating directly from the stem were counted for each treatment. Shoot length (cm) was measured from the stem base to the tip of the shoot. Root length (cm) was measured from stem to root tip.

### 4.4. Biochemical Analysis

#### 4.4.1. Methanolic Extract preparation for Estimation of Total Phenols Concentration (TPC), Total Flavonoids Concentration (TFC), and Antioxidant Activity

Potato shoots (2 g sample) were selected at random, and extraction was done according to [44]. Then, 20 mL of 80% methanol was mixed with the sample and shaken at 150 rpm for 12 h and was then filtered through Whatman filter paper No. 1 at room temperature. 

#### 4.4.2. Total Phenol Concentration (TPC) Estimation

The TFC was measured following Hoff and Singleton (1977) [63]. A mixture of 50 µL methanolic extract, 100 µL of Folin-Ciocalteu reagent and 850 µL of methanol was prepared and held at 23 + 1 °C for 5 min. Sodium carbonate (20% *w*/*v*) was then added to the mixture and kept for 30 min to react. The TPC absorbency was measured at 750 nm. The results were expressed in g.kg^−1^. The fresh weight (FW) gallic acid equivalent, after quantification from the gallic acid absorbance calibration curve, was at known concentrations. 

#### 4.4.3. Total Flavonoids Concentration (TFC) Estimation

The TFC was measured by a revised colorimetric method as described by Zhishen et al. (1999) [64]. A measure of 250 µL of methanolic extract was mixed with 1.25 mL of water and 75 µL with 5% *w*/*v* of NaNO_2_. The solution was held for 6 min before being mixed with 150 µL 10% *w*/*v* of AlCl_3_, 0.5 mL of NaOH (1 M), and 275 µL of distilled water. Absorbance at 510 nm was recorded for the total flavonoids. The calibration curve was obtained from the absorbance of known concentrations of catechin for the quantification of total flavonoids and the results were given as the g.kg^−1^ FW catechin equivalent. 

#### 4.4.4. Antioxidant Capacity by DPPH Radical Scavenging Assay

The methanolic extract of the in vitro potato shoots was analyzed for free-radical scavenging activity in DPPH (1,1-diphenyl-2-picrylhy-drazyl) methanol [65]. An amount of 0.1 mL of methanolic extract and 0.9 mL of fresh DPPH methanol solution (0.1 mM) was mixed. As a control, the same quantity of methanol was used. After dark incubation at an ambient temperature for 30 min, the absorbance was noted at 517 nm. The percentage of scavenging activity was calculated by the formula:

DPPH radical scavenging (%) = [(Abs control − Abs sample)/Abs control] × 100(1)

The dose-response curves were used to calculate IC_50_ (half-maximal inhibition concentration) values. 

#### 4.4.5. Enzymes Activity Evaluation

A crude extract of enzymes was prepared by homogenizing 1 g of shoot sample with tris-HCl (20 mM) with a pH buffer of 7.2 that was then centrifuged at 10,000 rpm for 10 min at 4 °C [66]. The supernatant was stored at −20 °C prior to the peroxidase (POD) and polyphenol oxidase (PPO) assays.

#### 4.4.6. Peroxidase (POD) Assay

The peroxidase (EC 1.11.1.7) activity was examined as described by Mar’ia and Cascone (1995) [67] and Awad et al. (2017) [66]. The reaction mixture was composed of 1000 µL:10 µL of H_2_O_2_ (0.97 M), 80 µL of guaiacol (0.5 M), 250 µL of sodium acetate with a buffer (pH 5.5), and 50 µL of crude extract. After allowing one minute for guaiacol oxidation, absorbance at 470 nm was recorded. Per unit activity of enzyme is the quantity of enzyme required for 1.0 O.D. min^−a1^ change under the standard assay conditions.

#### 4.4.7. Polyphenol Oxidase (PPO) Assay

Polyphenol oxidase (EC 1.14.18.1) activity was examined by catechol substrate following the methodology of Jiang et al. (2002) [68]. A measure of 200 µL of crude extract was mixed with 2800 µL of catechol (20 mM) solution in 0.01 M sodium phosphate with a buffer (pH 6.8). Absorbance increased at 400 nm and was recorded over 3 min. Results are expressed as per unit activity of enzyme required for 0.1 O.D. min^−1^ change under the standard assay conditions.

### 4.5. Statistical Analysis of Data

The experiment was of a completely randomized factorial design. There were two varieties, 5 LiCl and 5 mannitol concentrations with 3 replications per treatment. The results were analyzed by analysis of variance (ANOVA) using the software Statistix (version 8.1, Analytical Software, Tallahassee, FL, USA). The least significant difference (LSD) was used to separate differences between treatments at *p* < 0.05. Prism 8 (GraphPad Software, San Diego, CA, USA) was used for plotting data.

## 5. Conclusions

In summary, the two cultivars responded differently to LiCl and mannitol simulated ionic and osmotic stress. A morphological and biochemical analysis of both cultivars suggests that the cultivar Spunta may have a higher tolerance capability than BARI-401 based on the ability of Spunta to maintain growth parameters at higher LiCl and mannitol concentrations than BARI-401. In vitro screenings of potato genotypes, such as in this report, may help to provide a further understanding of novel abiotic stress mechanisms of tolerance and assist in the selection of genotypes with improved abiotic stress tolerance. Further open-field experimentation with controlled drought and salinity stress is required to validate the differences in performance of these two varieties in an agronomical setting.

## Data Availability

The data presented in this study are openly available in FigShare, doi:10.6084/m9.figshare.14423387.
